# Lacerated and Contused Wound—Treatment of

**Published:** 1869-06-15

**Authors:** C. F. Hart

**Affiliations:** Chicago


### *Lacerated and Contused Wound—Treatment of.*

BY C. F. HART, M.D., CHICAGO.

Was called Saturday, about half-past four o'clock, P. M., of March 27th, to 99 W. Polk street, to see Mr. L.'s son, age between 12 and 13 years, who had been caught in the machinery of one of the planing mills near by.

I found him suffering from a wound in the thighs and nates, extending as high as the lumbar region, the right leg posteriorly, and inwards, to the inner edge (if I may so express myself), outward, forward and inward, to the sartorius muscle, where the femoral artery passes under it, downwards as much as two-thirds the length of the thigh. The right hand was also caught, taking off a part of the adductor muscle of the thumb, and extending up the wrist, about one and a half inches, and the same in width, exposing the tendons, but not injuring them.

Dr. Wanzer was called and reached there before any thing was done more than ordering the dressing. We both thought it rather a hopeless case, and the probabilities were that he would not recover from the shock. Chloroform was administered, and we made a more thorough examination, and found the following parts destroyed, or partially so. The muscles were the gluteus maximus, both sides, that of the right side from near its middle to its insertion, gluteus medius, internal and external sphincters, all gone, with surrounding adipose tissue, biceps, tensor vaginis femoris, rectus, and touching the sartorius. The arteries were superficial branches of the gluteus, external hemorrhoidal, and external circumplex. The fascia on the anterier of the thigh was not divided but badly contused, and torn loose, so that you could pass your hand under nearly to the ilium, with the muscles torn apart from below the trochanter, making a hole three or four inches in length. The nerves were all the superficial of those parts, viz.: branches of the great and small sciatic, the gluteal and hemorrhoidal, the latter being a branch of the pudic. The great sciatic was exposed from one to one and a half inches above where it passes under the biceps, but not injured.

What was to be done? Amputation was not only impracticable, but impossible. We decided to try to save both life and limb. After removing the clothes and chips, the parts were well cleansed with tepid aqua, then washed with a solution of carbolic acid, composed of one part acid, thirty parts aqua, then anointed with carbolic oil-acid—Carbol. ℨi, Ole. Lini. ℥vi. Carbolic paste was then applied over the entire wound, from one-eighth to one-fourth of an inch thick. Calcis Carbonas, Ole. Acid Carbol., q. s. Make in soft paste. He rallied well from the chloroform, pulse about fifty, and compressed. Stimulants were administered, and directed to be continued every twenty or thirty minutes until my return.

9 o'clock P. M. Had slept nearly a half hour. Pulse about 60. Gave him Tinct. Opii, Camph., gtt. xx.

Sunday, half-past six A. M. Had vomited twice during the night, and slept very little, suffering a good deal of pain. Pulse 80. Morphia Sulph. gr. $\frac{1}{8}$, and repeat every two hours.

10 A. M.—Morphia vomited him every time he took it. Stomach very irritable. Discontinued Morph., and gave Tinc. Aconite Rad. gtt. ij, aqua ℥viij. Dose ℥ij. every half hour.

2½ P. M.—Still vomiting. Pulse 90, quick and small. Gave Calcis aqua ℥ij., milk, fresh, ℥ij., and continue every hour if necessary.

6 P. M.—Vomiting ceased after one dose Calcis aqua. Pulse 104, soft. Had slept some. Continue Aconite every two hours.

Monday, 8½ A. M.—Slept a little during the night, but not refreshed from it. Has a weak, debilitated appearance. Free of pain; pulse 140, small and quick. Continue Aconite every hour. Tinc. Ferri Mur. gtt. x every three hours.

12 M.—Still easy. Has had short naps, but does not seem rested from them. Pulse 124, soft. Continue Aconite and Ferri, as before. Opii Pulvis grs. x. M. et Div. in pill xx., one every four hours.

4. P. M.—Has had one hour's sleep; resting easy; pulse 124, soft.

7 P. M.—No change.

Tuesday, 8 ½ A. M.—Had a good night's sleep. Seems much refreshed and strengthened from it. Dressed wounds; hand and wrist doing well; thigh and nates sloughing, with appearance of gangrene. Syringed the parts with aqua tepid. Applied Ole. Carbol. freely, and covered well with Carbolic paste. Pulse small and quick, 140. Directed stimulants freely and heat to the feet.

12 M.—Pulse 150, quick and small; eyes glassy; respiration hurried; great restlessness, with shooting pains through the wounds. Continue stimulants, Ferri and Aconite as before.

3 P. M.—Not much change. Seems to rest easy for a few minutes after each dose of the aconite. Continue it every half-hour, as in the commencement.

6 P. M.—Pulse full and soft, 140, still the shooting pains through the wounds. Continue aconite every hour.

9 P. M.—Has rested tolerably well since last visit. Pulse about the same.

Wednesday, 9 A. M.—Has had a good night's sleep, and is much refreshed. Ate seven or eight oysters, and relished them. Wounds again dressed. Granulations sat up under the gangrenous parts; not much fatigued from the dressing. (Anæsthetics not used in dressing.) Changed him from a feather to a straw bed.

2 P. M.—Resting comfortably; slight pains in the wounds, with some trouble about micturating, as if there were stricture. Has to strain some time before the water starts. Pulse about the same.

4 P. M.—Was sent for. Is suffering great pain through the bowels, especially the hypogastric region. Great restlessness, with half-sighing and moaning. Pulse 120, small. Directed half-grain Opii and hot humulus fermentations to the bowels.

6½ P. M.—Still suffering severely from the bowels. Pulse the same. Passed some urine, thick, turbid, and small quantity, causing great pain and straining. Directed Spts. Nitri Dulc. ℨss., Potass, Nitras, Sol. Sat. ℨi every hour and repeat Opii every two hours.

11½ P. M.—Has passed his urine once since last visit, with some pain and straining. Still restless; great tenderness over the hypogastric and umbilical regions. Changed Opii to Morphia grs. ⅛ every two hours, or until he gets easy.

Thursday, 9 A. M.—Rested tolerably well during the latter part of the night. Passed his water three times since last visit, without pain and clear. Took some oyster soup about 4 o'clock A. M., after it some Vini Gallici. At 6 A. M. ate a plate full of porridge, and called for more. Pains subsiding. Very much prostrated from the night's suffering. Pulse 106, soft. On examining the wounds we found the granulations in the arm in a very languid condition, and gangrene had extended on the front part of the thigh to near the ilium and scrotum, and we were fearful it had reached the bowels. There was no tympanitis present, but the parts were exceedingly tender to the touch. He was too much exhausted to dress his wound, so we directed Vini Gallici, ad libitum, Morphia Sulph. grs. $\frac{1}{6}$ every two hours.

12 M.—Still very weak, and if any change, for the worse.

2½ P. M.—Still sinking. Pulse about 125 or 130. Respiration more hurried.

6½ P. M.—Gradually failing.

11½ P. M.—Still sinking. Pulse 130, weak and fluttering. Respiration more hurried. Voice rapidly failing. Can speak only in a whisper. Tongue and lips dry. Has suffered very little pain since morning. Has had two or three short naps. I should have stated before that during the afternoon and evening of yesterday the patient became anxious to have the family physician called. From some cause he had taken a dislike to myself and Dr. Wanzer, and I urged the family to send for him. His presence would quiet him more than an anodyne from us, and this morning Dr. Wanzer requested to be released from the consultations, and did withdraw.

Friday, 8½ A. M.—From about 1 to 6 A. M. has rested well and slept soundly. Pulse 120, soft, with considerable volume. Gangrene checked; the line of demarkation clearly set up. Dressed wound. Find the entire surface covered with granulations (being absent at this dressing, the treatment was changed); dressed with fresh lard.

12 M.—Has rested well since morning Slight pains in the wound; nothing unusual. Pulse 106, full and soft.

3 P. M.—Pulse the same, and resting well. Continue stimulants, etc., with occasionally the morphia.

6¼ P. M.—Resting easy; pulse 104, soft.

10½ P. M.—About the same.

Saturday, 8½ A. M.—Has had another tolerably good night's rest, though suffering, this morning, a good deal from his wounds, which were redressed. The gangrenous parts, most of them, are detached, or nearly so, a great deal of which was now removed. Fine, healthy pus, with granulations under the parts, but presenting a languid appearance. Dressed this morning with Unguent Basil, one part, fresh lard about four parts. Not approving of the treatment my connection with the case here ceased, though I consider the boy out of danger.

*April 5, 1869.*

